# Efficacy and safety of filgotinib in patients with moderately active rheumatoid arthritis and an inadequate response to methotrexate

**DOI:** 10.1093/rheumatology/keae486

**Published:** 2024-09-27

**Authors:** Maya H Buch, David Walker, Christopher J Edwards, Jane Barry, Laura Akroyd, Edmund V Ekoka Omoruyi, Peter C Taylor

**Affiliations:** Centre for Musculoskeletal Research, Division of Musculoskeletal and Dermatological Sciences, School of Biological Sciences, Faculty of Biology, Medicine & Health, University of Manchester, and NIHR Manchester Biomedical Research Centre, Manchester University NHS Foundation Trust, Manchester, UK; Department of Rheumatology, Northumbria Healthcare NHS Foundation Trust, North Shields, UK; Department of Rheumatology and NIHR Southampton Clinical Research Facility, University Hospital Southampton NHS Foundation Trust, Southampton, UK; Medical Affairs Inflammation, UK & Ireland, Galapagos Biotech Ltd, Uxbridge, UK; Medical Affairs Inflammation, UK & Ireland, Galapagos Biotech Ltd, Uxbridge, UK; Biostatistics, Galapagos NV, Mechelen, Belgium; Botnar Research Centre, Nuffield Department of Orthopaedics, Rheumatology and Musculoskeletal Sciences, University of Oxford, Oxford, UK

**Keywords:** clinical trials and methods, DMARDs, immunosuppressants, inflammation, JAK inhibitors, quality of life, rheumatoid arthritis

## Abstract

**Objectives:**

Clinical trials restricted to moderately active RA are limited. Filgotinib is approved for treating moderate to severe active RA. This *post hoc* analysis assessed the efficacy and safety of filgotinib in moderately active RA.

**Methods:**

In FINCH 1, patients with active moderate to severe RA and inadequate response to methotrexate received filgotinib 200 mg or 100 mg (FIL200/FIL100) once daily, adalimumab 40 mg every 2 weeks or placebo, all with methotrexate (*N* = 1755). This subgroup analysis was conducted in patients with a moderate baseline Disease Activity Score in 28 joints using C-reactive protein [DAS28-CRP; >3.2 to ≤5.1; *n* = 425 (24.2%)].

**Results:**

A higher proportion of patients achieved DAS28-CRP <2.6, Clinical Disease Activity Index (CDAI) remission (≤2.8), low disease activity (LDA) (DAS28-CRP ≤3.2 or CDAI ≤10) and American College of Rheumatology (ACR20/50/70) responses with FIL200 and FIL100 *vs* placebo at weeks 12 and 24. Week 12 ACR20 response rates (primary end point) were 77.9%, 67.8% and 43.8%, respectively. A total of ∼75% of patients achieved DAS28-CRP LDA by week 24 with either filgotinib dose. FIL200 and FIL100 elicited greater improvements in patient-reported outcomes than placebo. The efficacy of filgotinib, maintained through week 52, was comparable to that of adalimumab. Frequency of adverse events (AEs) was similar with filgotinib and adalimumab. Infections were the most common AEs; incidence rates were 40–53% in active treatment groups.

**Conclusion:**

In this subpopulation with moderately active RA, the efficacy and safety of filgotinib were similar to those in the overall FINCH 1 population (patients with active moderate to severe RA).

**Trial registration:**

ClinicalTrials.gov, http://clinicaltrials.gov, NCT02889796.

Rheumatology key messages

- FINCH 1 patients with moderately active RA were assessed in a *post hoc* subgroup analysis.
- Filgotinib 200 mg/100 mg elicited greater improvements in disease activity and patient-reported outcomes than placebo.
- Efficacy and tolerability results were similar to those for the overall study population.

## Introduction

Addition of an advanced therapy is recommended for the management of patients with RA after an inadequate response to one [American College of Rheumatology (ACR) [1, 2] and European Alliance of Associations for Rheumatology (EULAR) [1, 2]] or two or more [UK National Institute for Health and Care Excellence (NICE) [3–6]] conventional synthetic (cs)DMARDs. Advanced therapies include biologic (b)DMARDs and targeted synthetic (ts)DMARDs, e.g. Janus kinase (JAK) inhibitors. Risk factors for cardiovascular events and malignancies should be considered when selecting JAK inhibitors [2].

This escalation strategy is based on a treat-to-target approach, with the aim of achieving long-term remission or low disease activity (LDA) [1–6]. Currently, a considerable proportion of patients fail to reach this target, including those with moderately active RA [7–10]. Therefore, an increasing number of patients have persistent moderately active disease, which is associated with poor long-term outcomes, including structural damage, physical disability, low quality of life, and pain [7].

A limited number of studies in RA have focused on moderately active disease [11–15]. Until 2021, NICE guidance restricted advanced therapies to patients with high disease activity [Disease Activity Score in 28 joints using C-reactive protein (DAS28-CRP) >5.1] [16]. Filgotinib became the first advanced therapy to be recommended by NICE for treatment of patients with active moderate RA (DAS28-CRP >3.2 to ≤5.1) [4], followed by upadacitinib [5], adalimumab, etanercept (all as monotherapy/with methotrexate [MTX]) and infliximab (with MTX) [6]. These therapies are recommended for patients with an inadequate response to intensive therapy with ≥2 csDMARDs, including MTX.

Filgotinib is a JAK1-preferential inhibitor approved for treatment of patients with moderate to severe active RA who have responded inadequately, or are intolerant, to ≥1 DMARDs [17]. The approval was based on results from the Phase 3 FINCH 1–3 trials of filgotinib in patients with active RA and inadequate response to MTX or bDMARDs (filgotinib with MTX or csDMARDs) or those who were MTX naive (filgotinib with MTX/as monotherapy) [18–20]. Overall, the FINCH studies demonstrated that filgotinib reduced signs and symptoms of RA, improved physical function, inhibited progression of joint damage and was well tolerated.

The aim of this *post hoc* analysis was to assess the efficacy and safety of filgotinib in combination with MTX in the subgroup of patients from the FINCH 1 trial with moderately active RA and an inadequate response to MTX, based on DAS28-CRP >3.2 to ≤5.1 at baseline. This is particularly relevant to RA management in the UK, given the 2021 NICE guidance [4].

## Methods

### Study design

The methodology for FINCH 1 (NCT02889796) has been reported previously [18]. The study was a double-blind, 52-week, global Phase 3 trial in which patients with moderate to severe active RA and an inadequate response to MTX were randomized (3:3:2:3) to oral filgotinib 200 mg or 100 mg (FIL200/FIL100) once daily, subcutaneous adalimumab 40 mg every 2 weeks or placebo, all with stable background MTX. At week 24, placebo-treated patients were re-randomized (1:1) to FIL200 or FIL100 and continued MTX.

This manuscript reports a *post hoc* analysis of the FINCH 1 study and, as such, no specific ethics committee approval was sought or required. The manuscript reporting primary endpoints of the study was published in 2021 [18]. The trial was conducted in accordance with the Declaration of Helsinki and International Council for Harmonisation guidelines. The trial protocol was approved by the institutional review board or ethics committee at each study site. Safety data were reviewed by an independent monitoring committee; all potential major cardiovascular adverse events (MACEs) were independently adjudicated.

### Study population

All participants met the 2010 ACR/EULAR criteria [21] and provided written informed consent. This exploratory subgroup analysis was conducted in patients with moderate RA disease activity (DAS28-CRP >3.2 to ≤5.1 at baseline).

### Endpoints and assessments

The primary end point was the proportion of patients achieving a 20% improvement in ACR criteria (ACR20) at week 12 [18]. For this analysis, the proportion of patients achieving DAS28-CRP <2.6 and ≤3.2 [22], Clinical Disease Activity Index (CDAI) ≤2.8 [23] and ≤10 [24], and a 20%/50%/70% improvement in ACR criteria (ACR20/50/70) [25] was assessed at weeks 12, 24 and 52; duration of response was evaluated. Change from baseline in van der Heijde-modified total Sharp score (mTSS) [26] was assessed at weeks 12, 24 and 52, while patient-reported outcomes (PROs) were assessed at weeks 2, 4, 8, 12, 24 and 52 [Health Assessment Questionnaire–Disability Index (HAQ-DI) [27, 28], patient-reported pain] or at weeks 4, 12, 24 and 52 [Functional Assessment of Chronic Illness Therapy–Fatigue (FACIT-Fatigue) [29] and the 36-item Short-Form (SF-36) Physical Component Summary (PCS) and Mental Component Summary (MCS) [30]].

Adverse events (AEs), coded according to the Medical Dictionary for Regulatory Activities (MedDRA) v22.0, were recorded, including positively adjudicated MACEs, unadjudicated thromboembolic events, and other AEs of interest. AEs of interest were identified using standardized MedDRA queries and sponsor-defined medical search terms. Infections were defined as all preferred terms in the infections and infestations system organ class.

### Statistical analyses

For this *post hoc* analysis, efficacy, PRO measures, baseline characteristics and AEs were assessed in all randomized patients with moderately active RA who received ≥1 dose of study drug. Efficacy and PRO assessments made after the study medication was stopped, or the patient switched to standard of care, were excluded.

Baseline characteristics and AEs were analysed descriptively {mean [standard deviation (SD)], frequencies}. Additional details for descriptive analyses are provided in Supplementary Data S1, available at *Rheumatology* online.

Binary endpoints (DAS28-CRP <2.6 and ≤3.2, CDAI ≤2.8 and ≤10, and ACR20/50/70) were assessed using the normal approximation method with a continuity correction for 95% confidence intervals (CIs); *P*-values were based on pairwise comparisons per visit of each filgotinib group *vs* the placebo groups using a logistic regression model with treatment group and stratification factors (geographic region, prior exposure to bDMARDs and presence of rheumatoid factor or anti-cyclic citrullinated peptide antibodies) as covariates. Non-responder imputation was utilized for patients who required rescue therapy or had missing outcome data. Duration of response was analysed using Kaplan–Meier estimates.

Continuous endpoints (mTSS, HAQ-DI, patient-reported pain, SF-36 PCS and MCS, and FACIT-Fatigue) were assessed using a mixed-effects model for repeated measures (MMRM), with treatment, visit, treatment by visit interaction, stratification factors and baseline value as fixed effects and patient as a random effect. An unstructured variance-covariance matrix was assumed for the repeated measures. Least-squares (LS) means, 95% CIs and *P*-values were taken from the MMRM. Missing change scores were not imputed.

All analyses were exploratory and performed without multiplicity adjustment; nominal *P*-values are reported. Statistical analyses were performed using SAS v9.4 (SAS Institute, Inc., Cary, NC, USA).

## Results

### Baseline characteristics

Of the 1755 patients who received study drug in FINCH 1, 425 (24.2%) had moderate disease activity (DAS28-CRP >3.2 to ≤5.1) at baseline (Supplementary Table S1, available at *Rheumatology* online), with similar proportions (21.9–26.9%) across treatment groups. Baseline demographics were balanced across treatment arms (Table 1). Overall, the mean (SD) age was 52 (13.1) years, and most patients [*n* = 329 (77.4%)] were female. Baseline disease characteristics were similar across treatment groups, with an overall mean (SD) duration of RA of 7.8 (7.7) years and mean (SD) DAS28-CRP of 4.6 (0.42). Almost half of patients had received ≥2 prior csDMARDs [*n* = 209 (49.2%)] or concurrent corticosteroids [*n* = 203 (47.8%)].

**Table 1.** Baseline demographics and disease characteristics for patients with moderately active RA

	FIL200 (*n* = 104)	FIL100 (*n* = 121)	ADA (*n* = 72)	PBO (*n* = 128)	Total (*N* = 425)
Female, *n* (%)	79 (76.0)	93 (76.9)	57 (79.2)	100 (78.1)	329 (77.4)
Age, mean (SD), years	52 (13.5)	52 (13.1)	54 (13.9)	51 (12.3)	52 (13.1)
Weight, mean (SD), kg	70.2 (17.9)	69.2 (17.3)	69.4 (16.9)	68.9 (16.4)	69.4 (17.1)
Body mass index, mean (SD), kg/m^2^	26.2 (5.4)	25.5 (5.4)	26.1 (5.5)	26.0 (5.6)	25.9 (5.5)
Race, *n* (%)					
American Indian or Alaska Native	5 (4.8)	3 (2.5)	2 (2.8)	8 (6.3)	18 (4.2)
Asian	29 (27.9)	34 (28.1)	21 (29.2)	50 (39.1)	134 (31.5)
Black or African American	1 (1.0)	0	2 (2.8)	0	3 (0.7)
Native Hawaiian or Pacific Islander	0	0	0	1 (0.8)	1 (0.2)
White	69 (66.3)	81 (66.9)	47 (65.3)	68 (53.1)	265 (62.4)
Other	0	3 (2.5)	0	1 (0.8)	4 (0.9)
Duration of RA from diagnosis, mean (SD), years	7.9 (7.6)	8.6 (8.4)	6.8 (6.6)	7.6 (7.5)	7.8 (7.7)
hsCRP, mean (SD), mg/L	6.8 (9.9)	6.2 (9.2)	6.2 (8.1)	5.4 (6.3)	6.1 (8.4)
RF positive, *n* (%)	78 (75.0)	85 (70.2)	50 (69.4)	92 (71.9)	305 (71.8)
Anti-CCP positive, *n* (%)	87 (83.7)	98 (81.0)	58 (80.6)	106 (82.8)	349 (82.1)
RF and anti-CCP positive, *n* (%)	73 (70.2)	81 (66.9)	46 (63.9)	86 (67.2)	286 (67.3)
bDMARD naive, *n* (%)	100 (96.2)	116 (95.9)	70 (97.2)	128 (100.0)	414 (97.4)
1 prior csDMARD, *n* (%)	51 (49.0)	66 (54.5)	37 (51.4)	62 (48.4)	216 (50.8)
≥2 prior csDMARDs, *n* (%)	53 (51.0)	55 (45.5)	35 (48.6)	66 (51.6)	209 (49.2)
Concurrent corticosteroid use on day 1, *n* (%)	52 (50.0)	59 (48.8)	27 (37.5)	65 (50.8)	203 (47.8)
Dose, mg/day	6.2 (2.4), *n* = 51	5.6 (2.8), *n* = 59	5.2 (1.6), *n* = 27	5.7 (2.4), *n* = 65	5.7 (2.4), *n* = 202
Concurrent methotrexate dose on day 1, *n* (%), mg/week	14.5 (5.3), *n* = 104	15.3 (4.8), *n* = 121	14.3 (5.2), *n* = 71	14.0 (4.2), *n* = 128	14.6 (4.8), *n* = 424
DAS28-CRP, mean (SD)	4.6 (0.43)	4.5 (0.47)	4.6 (0.37)	4.6 (0.37)	4.6 (0.42)
CDAI, mean (SD)	27.1 (6.0)	25.6 (5.9)	27.1 (5.2)	26.9 (5.2)	26.6 (5.6)
SJC66, mean (SD)	10 (4.0)	11 (5.6)	11 (4.9)	10 (4.1)	10 (4.7)
SJC28, mean (SD)	7 (3.0)	7 (3.1)	7 (3.0)	7 (2.8)	7 (3.0)
TJC68, mean (SD)	14 (6.5)	14 (6.6)	14 (5.9)	15 (7.6)	14 (6.8)
TJC28, mean (SD)	9 (3.7)	8 (3.4)	9 (3.5)	9 (3.5)	9 (3.5)
SGA score, mean (SD), mm	53 (20.2)	48 (19.7)	52 (20.3)	53 (18.8)	51 (19.7)
PGA score, mean (SD), mm	57 (16.0)	55 (16.6)	59 (16.8)	58 (16.4)	57 (16.4)
Pain score, mean (SD), mm	50 (22.4)	48 (20.8)	49 (20.9)	51 (19.0)	50 (20.6)
HAQ-DI, mean (SD)	1.2 (0.67)	1.1 (0.63)	1.2 (0.63)	1.2 (0.66)	1.2 (0.65)
mTSS, mean (SD)	27.7 (33.5), *n* = 102	29.9 (35.4), *n* = 120	20.1 (32.3), *n* = 70	33.1 (54.7), *n* = 127	28.7 (41.5), *n* = 419
SF-36 PCS score, mean (SD)	37.0 (8.2)	38.6 (8.1)	37.5 (8.6)	37.5 (7.3)	37.7 (8.0)
SF-36 MCS score, mean (SD)	46.0 (10.2)	47.5 (11.5)	45.1 (8.5)	45.4 (10.1)	46.1 (10.3)
FACIT-Fatigue score, mean (SD)	31.0 (10.4), *n* = 104	33.4 (9.7), *n* = 121	31.1 (9.5), *n* = 70	32.2 (8.8), *n* = 125	32.1 (9.6), *n* = 420

ADA: adalimumab; b/csDMARD: biologic/conventional synthetic DMARD; CCP: cyclic citrullinated peptide; CDAI: Clinical Disease Activity Index; DAS28-CRP: Disease Activity Score in 28 joints using C-reactive protein; FACIT-Fatigue: Functional Assessment of Chronic Illness Therapy–Fatigue; FIL100/200: filgotinib 100/200 mg; HAQ-DI: Health Assessment Questionnaire–Disability Index; hsCRP: high-sensitivity C-reactive protein; mTSS: van der Heijde-modified total Sharp score; P/SGA: Physician's/Subject’s Global Assessment of Disease Activity; PBO: placebo; RF: rheumatoid factor; SD: standard deviation; SF-36 P/MCS: 36-item Short-Form Physical/Mental Component Summary; S/TJCxx: swollen/tender joint count based on xx joints.

### Efficacy

#### DAS28-CRP <2.6 or CDAI remission, LDA and ACR response

**Figure 1.**
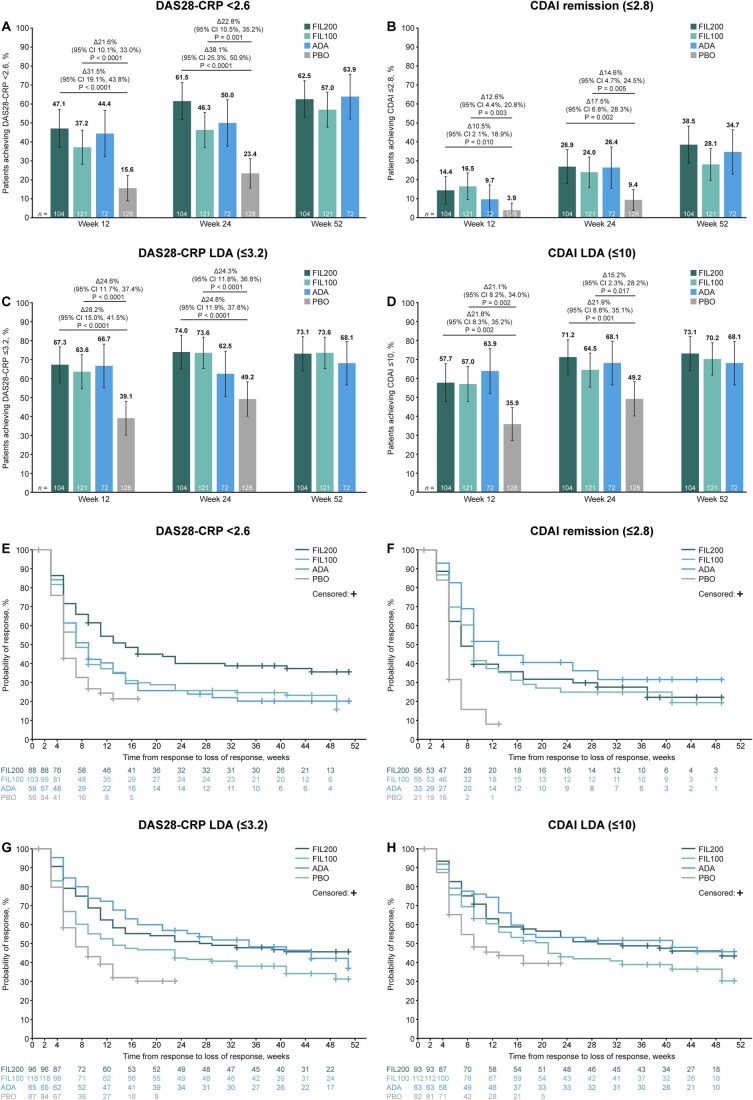
DAS28-CRP <2.6/CDAI remission/LDA in patients with moderately active RA up to week 52. (**A**–**D**) Error bars show 95% CIs. All *P*-values are nominal, not adjusted for multiplicity, and should be considered exploratory. (**E**–**H**) Non-responder imputation was utilized for patients who required rescue therapy or had missing outcome data. Duration of response was calculated as end of response to start of response +1. Δ: difference; ADA: adalimumab; CDAI: Clinical Disease Activity Index; CI: confidence interval; DAS28-CRP: Disease Activity Score in 28 joints using C-reactive protein; FIL100/200: filgotinib 100/200 mg; LDA: low disease activity; PBO: placebo.

**Figure 2.**
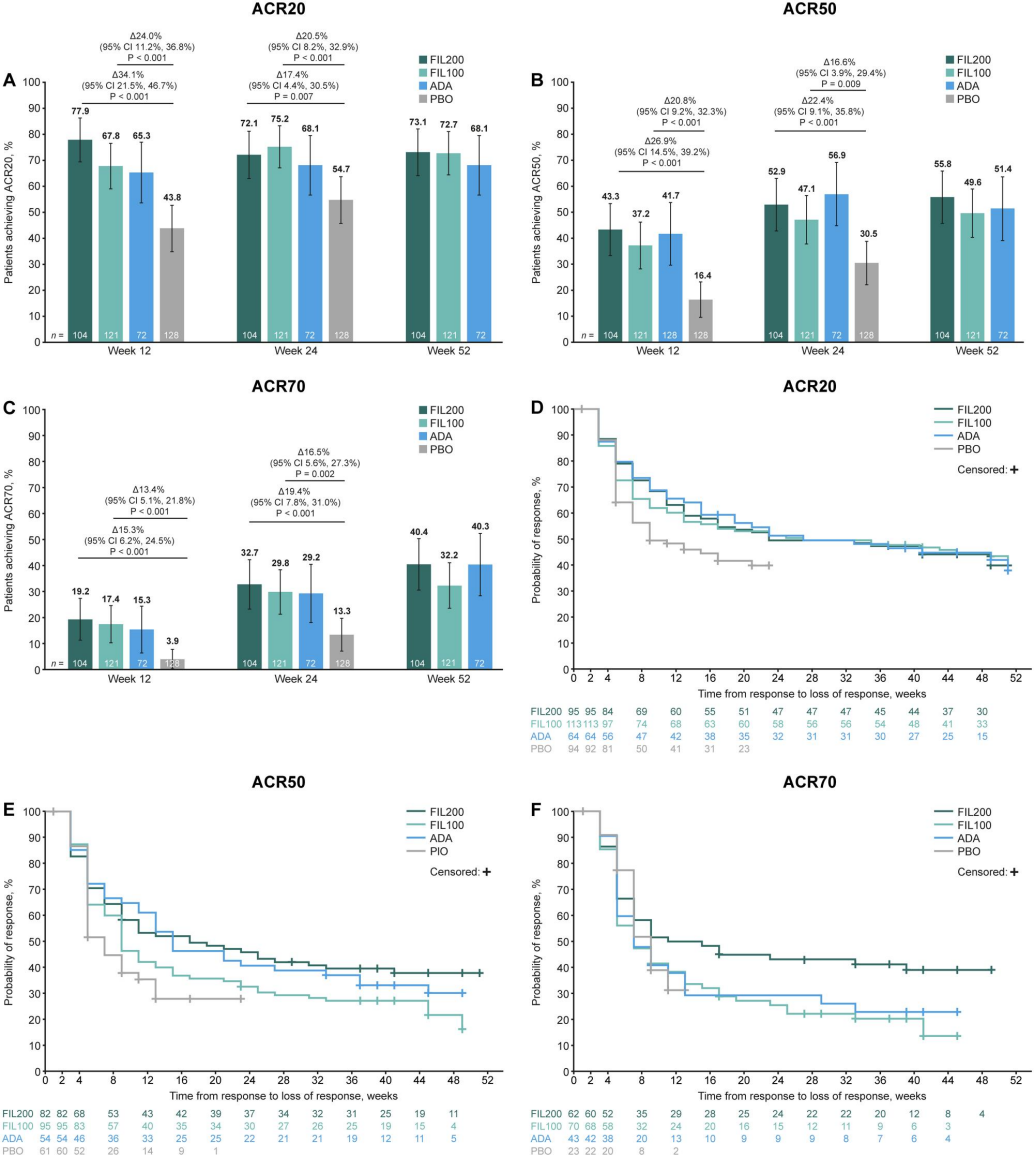
ACR20/50/70 in patients with moderately active RA up to week 52. (**A**–**C**) Error bars show 95% CIs. All *P*-values are nominal, not adjusted for multiplicity, and should be considered exploratory. (**D**–**F**) Non-responder imputation was utilized for patients who required rescue therapy or had missing outcome data. Duration of response was calculated as end of response to start of response +1. Δ: difference; ACR20/50/70: 20%/50%/70% improvement in American College of Rheumatology criteria; ADA: adalimumab; CI: confidence interval; FIL100/200: filgotinib 100/200 mg; PBO: placebo.

A higher proportion of patients achieved DAS28-CRP <2.6 or CDAI remission, LDA and ACR responses with either filgotinib dose than with placebo at weeks 12 and 24; response rates across these endpoints were generally comparable between adalimumab and filgotinib (either dose) at weeks 12, 24 and 52 (Figs 1A–D and 2A–C). FIL200 and FIL100 maintained efficacy through week 52 (Fig. 1A–D and 2A–C). An incremental benefit in DAS28-CRP <2.6 or CDAI remission, CDAI LDA, and ACR50/70 response was observed with filgotinib treatment over time (Figs. 1A, B and D and 2B–C).

The median duration of DAS28-CRP <2.6 was 15, 7 and 9 weeks, while that of CDAI remission was 7, 9 and 13 weeks, in the FIL200, FIL100 and adalimumab groups, respectively (Fig. 1E–F; Supplementary Table S2, available at *Rheumatology* online). For the placebo group, the median duration was 5 weeks for both DAS28-CRP <2.6 and CDAI remission. The median duration of DAS28-CRP LDA (28 *vs* 35 weeks) and CDAI LDA (33 *vs* 41 weeks) was comparable between FIL200 *vs* adalimumab (Fig. 1G–H; Supplementary Table S2, available at *Rheumatology* online). The median duration of response was 23, 27 and 27 weeks for ACR20; 17, 9 and 15 weeks for ACR50; and 11, 7 and 7 weeks for ACR70, in the FIL200, FIL100 and adalimumab groups, respectively (Fig. 2D–F; Supplementary Table S2, available at *Rheumatology* online). For the placebo group, the median duration was 9, 7 and 7 weeks for ACR20, 50 and 70, respectively.

#### Radiographic progression

**Figure 3.**
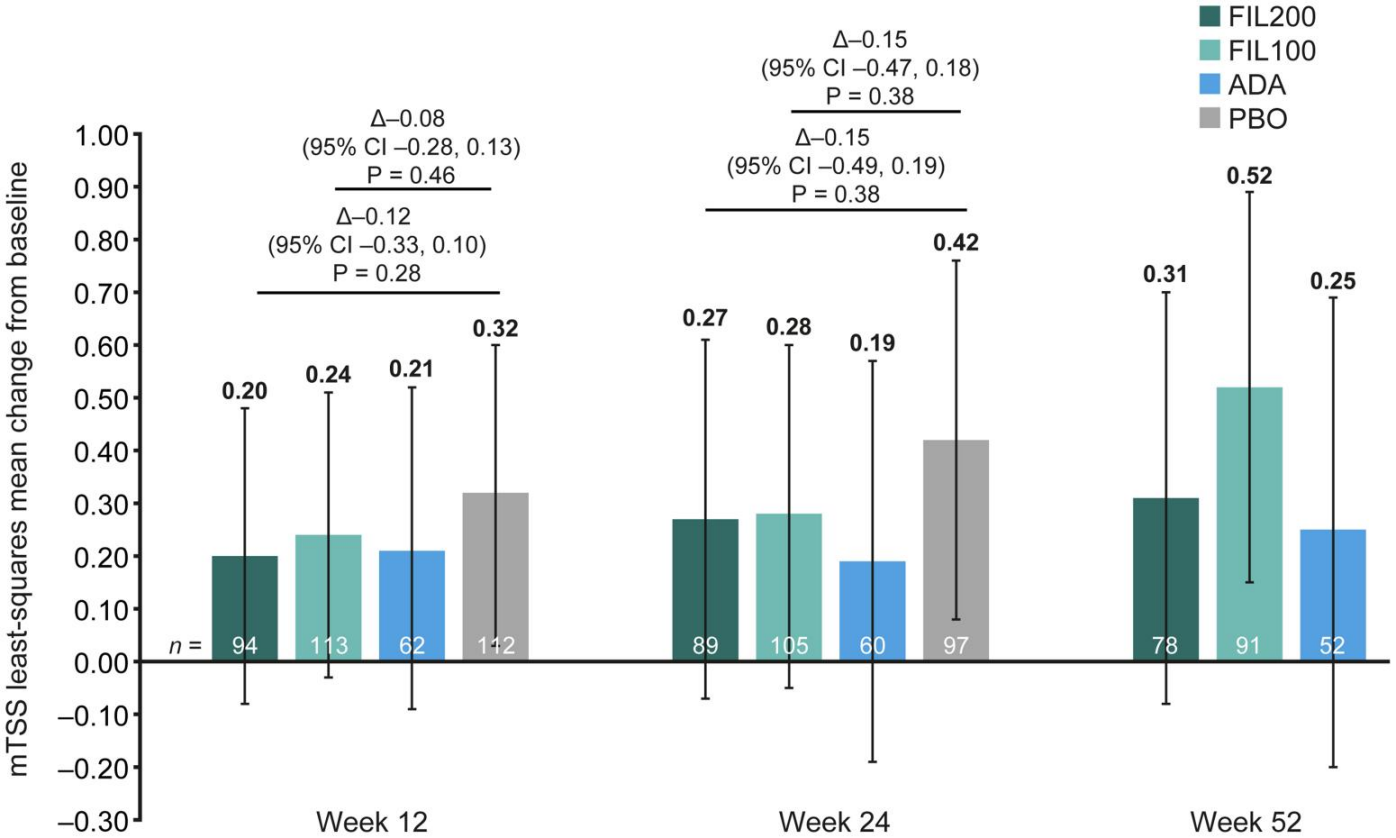
Radiographic progression up to week 52 in patients with moderately active RA. Error bars show 95% CIs. All *P*-values are nominal, not adjusted for multiplicity, and should be considered exploratory. Δ: difference; ADA: adalimumab; CI: confidence interval; FIL100/200: filgotinib 100/200 mg; mTSS: van der Heijde-modified total Sharp score; PBO: placebo.

Overall, radiographic progression was minimal. Based on point estimates, less radiographic progression was observed with filgotinib than with placebo; however, the 95% CIs largely overlapped [LS mean (95% CI) at week 24: FIL200, 0.27 (−0.07, 0.61); FIL100, 0.28 (−0.05, 0.60); placebo, 0.42 (0.08, 0.76); Fig. 3]. Radiographic progression was numerically similar between adalimumab and FIL200/FIL100 at weeks 12 and 24, and between adalimumab and FIL200 at week 52 (Fig. 3).

### Patient-reported outcomes

**Figure 4.**
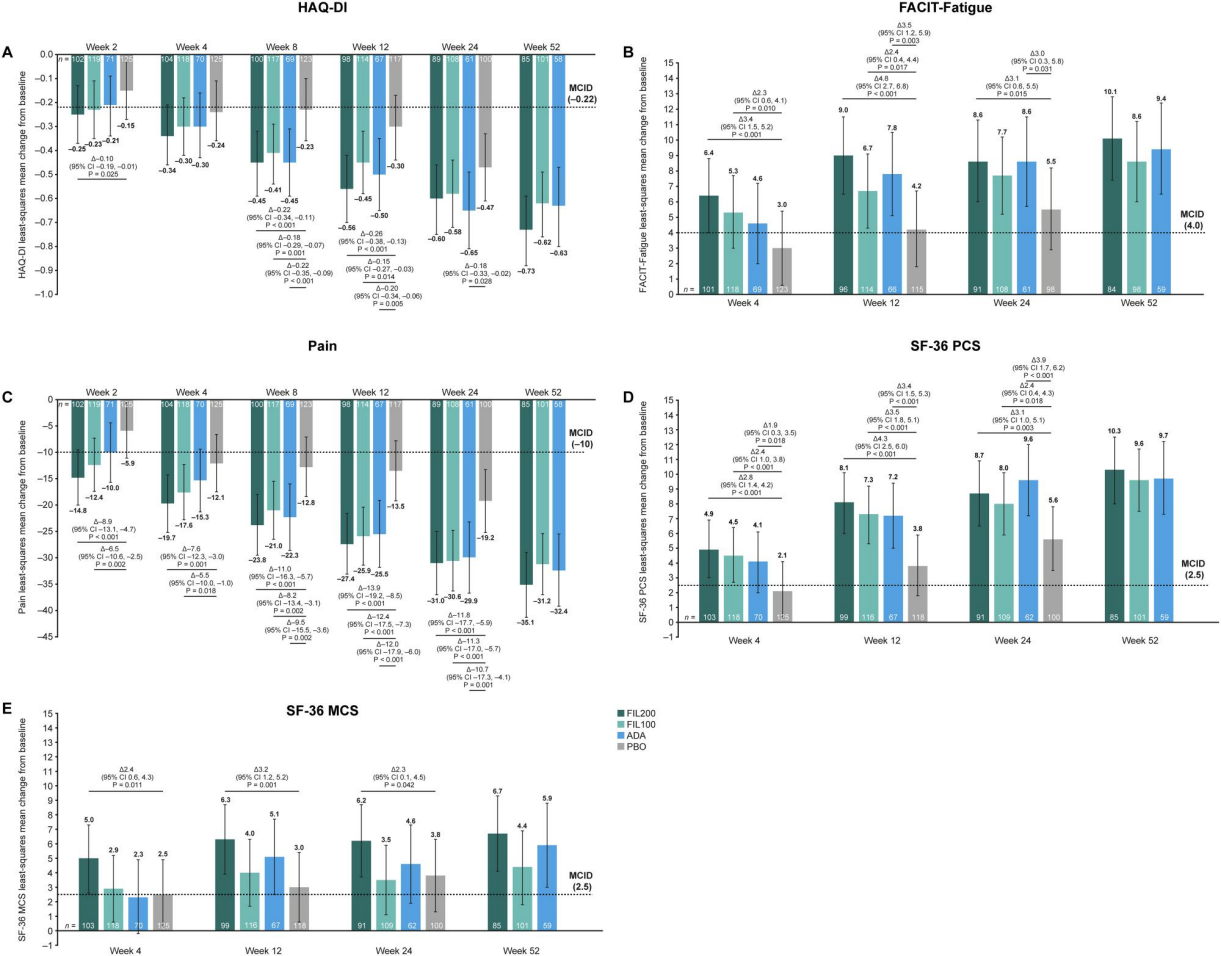
PRO improvements up to week 52 in patients with moderately active RA. Error bars show 95% CIs. All *P*-values are nominal, not adjusted for multiplicity, and should be considered exploratory. At each time point, only differences of *P* <0.05 are shown. Δ: difference; ADA: adalimumab; CI: confidence interval; FACIT-Fatigue: Functional Assessment of Chronic Illness Therapy–Fatigue; FIL100/200: filgotinib 100/200 mg; HAQ-DI: Health Assessment Questionnaire–Disability Index; MCID: minimal clinically important difference; PBO: placebo; PRO: patient-reported outcome; SF-36 P/MCS: 36-item Short-Form Physical/Mental Component Summary.

Changes in PROs from baseline and in relation to minimal clinically important differences (MCIDs) are shown in Fig. 4. Greater improvement in HAQ-DI was observed with FIL200 *vs* placebo at week 2 [LS mean (95% CI) change, −0.25 (−0.37, −0.13) *vs* −0.15 (−0.27, −0.03)] and with FIL200 and FIL100 *vs* placebo at week 8 [−0.45 (−0.59, −0.32) and −0.41 (−0.54, −0.29) *vs* −0.23 (−0.36, −0.10)] and week 12 [−0.56 (−0.70, −0.42) and −0.45 (−0.58, −0.32) *vs* −0.30 (−0.44, −0.17)]. Improvements in HAQ-DI were numerically higher with either filgotinib dose than with placebo at week 24. Greater improvement in HAQ-DI with adalimumab than with placebo was observed from week 8 through to week 24 (Fig. 4A).

Improvement in FACIT-Fatigue score was greater with FIL200/FIL100 *vs* placebo at week 4 [LS mean (95% CI) change, 6.4 (4.0, 8.8)/5.3 (3.0, 7.7) *vs* 3.0 (0.6, 5.4)] and week 12 [9.0 (6.5, 11.5)/6.7 (4.2, 9.1) *vs* 4.2 (1.8, 6.7)], and with FIL200 *vs* placebo at week 24 [8.6 (6.0, 11.3) *vs* 5.5 (2.9, 8.2)]. Greater improvements in FACIT-Fatigue score were observed with adalimumab than placebo at week 12 [7.8 (5.1, 10.5) for adalimumab] and week 24 [8.6 (5.7, 11.5)] (Fig. 4B).

Pain was improved with FIL200 and FIL100 relative to placebo from week 2 [LS mean (95% CI) change, −14.8 (−20.0, −9.5), −12.4 (−17.4, −7.3) and −5.9 (−11.1, −0.6), respectively] through to week 24 [−31.0 (−37.0, −25.0), −30.6 (−36.6, −24.8) and −19.2 (−25.2, −13.3), respectively]. Greater improvements in pain with adalimumab relative to placebo were observed from week 8 [−22.3 (−28.6, 16.0) for adalimumab] onwards (Fig. 4C). A numerically greater improvement in pain was observed at weeks 2 and 4 with FIL200 relative to adalimumab.

A greater improvement in SF-36 PCS score with FIL200, FIL100 and adalimumab relative to placebo was observed at week 4 [LS mean (95% CI) change, 4.9 (3.0, 6.9), 4.5 (2.7, 6.4), 4.1 (2.0, 6.1) and 2.1 (0.2, 4.1), respectively] through to week 24 (Fig. 4D). Improvements in SF-36 MCS score were observed with FIL200 relative to placebo from week 4 [5.0 (2.6, 7.3) and 2.5 (0.2, 4.9), respectively] through to week 24. Improvement in SF-36 MCS score with filgotinib (either dose) met the criterion for MCID from week 4 onwards, while adalimumab met this criterion from week 12 onwards (Fig. 4E).

For all PROs, improvement beyond the MCID was maintained through week 52 with FIL200 and FIL100 (Fig. 4). The improvement in HAQ-DI, pain and SF-36 PCS score increased over time with both filgotinib doses (Fig. 4A, C and D). In general, improvements in PROs were numerically comparable between adalimumab and filgotinib (either dose) at weeks 12, 24 and 52.

### Safety

Treatment-emergent AEs (TEAEs) are presented in Table 2. The incidence of TEAEs and serious TEAEs was similar across all active treatment arms throughout the 52-week study period. The frequency of Grade ≥3 TEAEs for patients receiving FIL200, FIL100 and adalimumab was 10.6% (*n* = 11), 9.9% (*n* = 12) and 5.6% (*n* = 4), respectively. The frequency of Grade ≥3 TEAEs for patients receiving placebo (weeks 0–24) was 4.7% (*n* = 6). Four deaths were reported: two in patients receiving FIL200 (1.9%; pneumonia and septic shock [*n* = 1] and multiple respiratory conditions, including infection, and *cor pulmonale* chronic [*n* = 1]), one in a patient receiving FIL100 (0.8%; myocardial infarction) and one during placebo on FIL100 period (0.8%; varicella).

**Table 2.** TEAEs up to week 52 for patients with moderately active RA

*n* (%)	Weeks 0–52			Weeks 24–52		Weeks 0–24
	FIL200 (*n* = 104)	FIL100 (*n* = 121)	ADA (*n* = 72)	PBO on FIL200 period (*n* = 40)	PBO on FIL100 period (*n* = 62)	PBO on PBO period (*n* = 128)
TEAEs	88 (84.6)	88 (72.7)	57 (79.2)	21 (52.5)	34 (54.8)	77 (60.2)
Grade ≥3	11 (10.6)	12 (9.9)	4 (5.6)	2 (5.0)	4 (6.5)	6 (4.7)
Serious	8 (7.7)	12 (9.9)	5 (6.9)	1 (2.5)	2 (3.2)	2 (1.6)
Leading to death	2 (1.9)	1 (0.8)	0	0	1 (1.6)	0
TEAEs of interest						
Infection	55 (52.9)	49 (40.5)	36 (50.0)	6 (15.0)	13 (21.0)	32 (25.0)
Serious	3 (2.9)	3 (2.5)	1 (1.4)	1 (2.5)	1 (1.6)	1 (0.8)
Grade ≥3	3 (2.9)	4 (3.3)	1 (1.4)	1 (2.5)	1 (1.6)	2 (1.6)
Herpes zoster infection	2 (1.9)	2 (1.7)	0	1 (2.5)	0	2 (1.6)
Grade ≥3	0	0	0	0	0	0
Malignancy (excluding NMSC)	1 (1.0)	1 (0.8)	1 (1.4)	0	0	0
MACEs	0	1 (0.8)	1 (1.4)	0	0	0
Unadjudicated thromboembolic events*	0	0	0	0	0	0

MACEs were positively adjudicated.

* Deep vein thrombosis or pulmonary embolism.

ADA: adalimumab; FIL100/200: filgotinib 100/200 mg; MACE: major adverse cardiovascular event; NMSC: non-melanoma skin cancer; PBO: placebo; TEAE: treatment-emergent adverse event.

Infections were the most common TEAEs. Overall, infections and serious infections occurred in a higher proportion of patients receiving filgotinib or adalimumab through to week 52 *vs* placebo up to week 24. The frequency of infections for patients in the FIL200 [*n* = 55 (52.9%)] and adalimumab ([*n* = 36 (50.0%)] arms was numerically higher than that for patients in the FIL100 [*n* = 49 (40.5%)] and placebo [*n* = 32 (25.0%)] arms. Infections that occurred in ≥3% of patients in the FIL200 arm were: nasopharyngitis [*n* = 15 (14.4%)], upper respiratory infection [*n* = 9 (8.7%)], urinary tract infection [*n* = 5 (4.8%)], influenza [*n* = 4 (3.8%)] and pharyngitis [*n* = 4 (3.8%)]. Details of individual serious infections and Grade ≥3 infections (all occurred in ≤4 patients) are provided in Supplementary Data S2, available at *Rheumatology* online. Herpes zoster infection occurred in two patients in each of the FIL200 (1.9%), FIL100 (1.7%) and placebo (1.6%) arms but was not reported in the adalimumab arm. No Grade ≥3 herpes zoster infections were reported. Malignancy (excluding non-melanoma skin cancer) was reported in three patients, one each in the FIL200 (1.0%), FIL100 (0.8%) and adalimumab (1.4%) arms. No unadjudicated thromboembolic events were reported. An adjudicated MACE occurred in one patient in each of the FIL100 (0.8%) and adalimumab (1.4%) arms; no adjudicated MACEs occurred in the FIL200 arm.

## Discussion

In this *post hoc* analysis of patients with moderately active RA in FINCH 1, ∼50% of patients had an inadequate response to ≥2 csDMARDs, as required by NICE for initiation of filgotinib [4]. The proportions of patients achieving DAS28-CRP <2.6 or CDAI remission, LDA and ACR responses were higher with filgotinib (either dose) than with placebo at weeks 12 and 24. Compared with adalimumab, the duration of FIL200 response was numerically longer for DAS28-CRP <2.6 and ACR70, numerically similar for ACR20/50 and numerically shorter for CDAI remission and LDA. Compared with placebo, there was a numerically greater reduction in the rate of radiographic progression with filgotinib, and a greater improvement in PROs. A numerically greater improvement in pain was observed at weeks 2 and 4 with FIL200 relative to adalimumab, demonstrating a fast onset of action in a key outcome that affects patients’ quality of life. The efficacy of both doses of filgotinib was maintained through week 52 and was generally numerically comparable to that of adalimumab.

Mean baseline DAS28-CRP was 4.6—at the higher end of the moderately active disease range (>3.2 to ≤5.1)—suggesting a high burden of disease and increased risk of poor clinical outcomes [31]. A recent systematic review identified several prognostic factors in patients with moderately active disease who were at higher risk of disease progression, including DAS28-CRP ≥4.2 [31]. The results presented here demonstrate the benefit of advanced therapies in this higher-risk population of patients with moderate RA disease activity.

Patients with moderately active RA experience adverse long-term clinical outcomes [32], including radiographic progression [33], pain, fatigue and associated mental health issues. Work productivity is also decreased, with caregivers taking paid/unpaid leave or reducing working hours, and outpatient attendances and diagnostic/monitoring tests considerably impacting healthcare resources, contributing to a substantial economic burden [34]. Therefore, it is essential to adequately treat this population, especially because starting treatment at lower levels of disease activity helps to achieve better outcomes [35]. Although NICE approved treatment with targeted synthetics and biologics for patients with moderately active RA in 2021 [4–6], a change in treatment practice is required to optimally implement this guidance, underscoring the importance of educating more broadly in this regard [36].

The present results indicate that the efficacy and safety of filgotinib in patients with moderately active RA were similar to those seen in the overall FINCH 1 population [18]. Notably, ∼75% of patients with moderately active RA achieved ACR20 response by week 24 when treated with either dose of filgotinib, with a similar proportion also improving from moderate to low disease activity. Although it can be more difficult to demonstrate a percentage improvement from a lower baseline disease activity, patients with moderate active RA are often more likely to reach remission or LDA treatment targets than those with severe disease [35].

A *post hoc* analysis of four Phase 3 trials of upadacitinib demonstrated improvements in efficacy (ACR20 response, LDA and clinical remission), radiographic progression, patient-reported functioning, and pain *vs* placebo in those with moderate RA activity (DAS28-CRP >3.2 to ≤5.1) and an inadequate response to bDMARDs and/or csDMARDs, although the treatment period was shorter (12/14/26 weeks) than for this analysis (52 weeks) [37]. These upadacitinib data, alongside our results, present compelling and independent evidence for the efficacy of JAK inhibitors for the treatment of patients with moderate RA.

Clinically meaningful improvements in disease activity and PROs observed with both filgotinib doses were maintained through week 52, with an incremental benefit in high-hurdle endpoints, including DAS28-CRP <2.6 or CDAI remission, CDAI LDA, ACR50/70 response, HAQ-DI, pain and SF-36 PCS score over time. A group-based multivariate trajectory model also suggested that, in patients receiving FIL200 in FINCH 1, those not achieving CDAI LDA (2.9–10) within 6 months could still benefit from continued treatment [38]. The current data, and the results from the trajectory model, emphasize the benefit of persisting with treatment after an initial improvement to achieve optimal therapeutic responses. In clinical practice, patients’ expectations should be managed by explaining the variation in response trajectory from the outset of filgotinib therapy, to optimize adherence and achievable outcomes, enhancing trust and encouraging patient empowerment [39].

The overall frequency of TEAEs and serious TEAEs was similar among patients receiving either dose of filgotinib or adalimumab. Adjudicated MACEs occurred at the same low rate in the FIL100 and adalimumab arms; no adjudicated MACEs occurred in the FIL200 arm. No occurrences of unadjudicated thromboembolic events were reported. Approximately double the rate of infections, both non-serious and serious, was reported for patients treated with FIL200 and adalimumab through week 52 than for those treated with placebo up to week 24. The frequency of herpes zoster infection was low and similar across the filgotinib and placebo arms; no patients reported herpes zoster infection in the adalimumab arm. Overall study population TEAE data for FIL100, FIL200, adalimumab and placebo up to week 24 have been reported previously [18]. These results demonstrate that the safety profile of filgotinib for patients with moderately active RA is similar to that previously reported for the overall FINCH 1 population [18], as well as in the Phase 3 FINCH 2 and 3 trials [19, 20] (although the shorter treatment period of 24 weeks in FINCH 2 reduced the number of reported TEAEs).

Patients receiving FIL100 had a lower infection rate than those receiving FIL200 or adalimumab. Although there are currently no studies with dose titration in the filgotinib clinical trial programme, the similar efficacy of both doses of filgotinib could be of interest in clinical practice for patients who develop AEs or have an increased risk of complications. In patients aged ≥65 years, with history of atherosclerotic cardiovascular disease or other cardiovascular risk factors, or with malignancy risk factors, JAK inhibitors should only be used if no suitable treatment alternatives are available [17]. JAK inhibitors should also be used with caution in patients with known venous thromboembolism (VTE) risk factors, other than cardiovascular or malignancy risk factors [17]. The recommended dose of filgotinib for adults aged ≥65 years, and those at increased risk of MACEs, malignancy and VTE, is 100 mg once daily; this may be escalated to 200 mg once daily in case of insufficient disease control, with the lowest effective dose to be used for long-term treatment [17]. The opportunity to optimize therapy in older patients is important, as the prevalence of RA increases with age [40].

Limitations discussed previously for the FINCH 1 study, such as excluding patients with prior bDMARD failure and administering placebo for only 24 weeks, are applicable to the current analysis [18]. As the study population includes patients from several geographic regions, it may not be reflective of patients with moderately active RA requiring advanced therapy escalation in the UK. Due to the *post hoc* nature of this analysis and lack of multiplicity adjustment, all conclusions reported here should be considered exploratory and differences between study arms treated with caution. The unusually large improvements in PROs, particularly HAQ-DI, between weeks 12 and 24 for patients receiving placebo present a challenge when trying to differentiate the effects of filgotinib. Lastly, although the numerical difference in radiographic progression, as assessed by LS mean (95% CI) change from baseline in mTSS, appears favourable, the smallest detectable change was not computed [41].

## Conclusions

In this *post hoc* analysis of the subgroup of FINCH 1 participants with moderately active RA and an inadequate response to MTX, greater improvements in disease activity were observed in patients receiving filgotinib (200 mg or 100 mg) *vs* placebo. Treatment with filgotinib also resulted in numerically lower radiographic progression, reduced functional deficit and improvements in pain and physical and mental health. The efficacy and safety of filgotinib observed in this analysis were comparable to those reported for the overall FINCH 1 population, providing evidence to support the consideration of filgotinib for use in patients with moderately active RA. Real-world studies are ongoing to further assess the long-term effectiveness and safety of filgotinib.

## Supplementary material 

Supplementary material is available at *Rheumatology* online.

## Supplementary Material

keae486_Supplementary_Data

## Data Availability

Anonymized individual patient data will be shared upon request for research purposes dependent upon the nature of the request, the merit of the proposed research, and the availability of the data and its intended use. The full data sharing policy for Gilead Sciences, Inc., can be found at https://www.gileadclinicaltrials.com/en/transparency-policy#DataSharing. The data sharing policy for Galapagos NV can be found at https://www.clinicaltrials-glpg.com/us/en/data-transparency.html.
