# Supplementary material for: Efficacy and safety of filgotinib in patients with moderately active rheumatoid arthritis and an inadequate response to methotrexate
Source: Rheumatology (Oxford). 2024 Sep 27;64(4):1661–71. doi: 10.1093/rheumatology/keae486 (PMC11963078; doi:10.1093/rheumatology/keae486)
Supplement: keae486_Supplementary_Data [file keae486_supplementary_data.pdf]

Efficacy and safety of filgotinib in patients with moderately active rheumatoid arthritis and an inadequate response to methotrexate

Supplementary information

Supplementary Data S1. Supplementary methods: descriptive analyses

Age was calculated from date of first study drug administration. Duration of rheumatoid arthritis (years) = (first dose date − date of initial diagnosis +1)/365.25. Imputation rule for incomplete initial diagnosis date: the first day of the month was used for missing day; January was used for missing month. A patient was counted for exposure for each prior medication.

Treatment-emergent adverse events (TEAEs) began on or after the study drug start date up to 30 days after permanent discontinuation of study drug. For re-randomized patients, TEAEs that started prior to the first dose date of filgotinib were allocated to the placebo-controlled period, and TEAEs that started on or after the first dose date of filgotinib were allocated to the re-randomized period. Multiple adverse events were counted only once per patient for each preferred term.

Supplementary Data S2. Supplementary results: serious and Grade ≥3 infections

Serious infections included: pneumonia (n = 3; one each in the FIL200, FIL100, and placebo switched to FIL200 groups); cellulitis (n = 2; one each in the FIL100 and adalimumab groups); and bronchitis (FIL200), gastroenteritis (FIL200), infectious pleural effusion (placebo), pneumonia fungal (placebo), septic shock (FIL200), urinary tract infection (FIL100) and varicella (placebo switched to FIL100 group) (all n = 1).

Grade ≥3 infections included: pneumonia (n = 3; one each in the FIL200, FIL100, and placebo switched to FIL200 groups); cellulitis (n = 2; one each in the FIL100 and adalimumab groups); and bronchitis (FIL200), epididymitis (FIL200), infectious pleural effusion (placebo), nasopharyngitis (placebo), pneumonia fungal (placebo), septic shock (FIL200), upper respiratory tract infection (FIL100), urinary tract infection (FIL100) and varicella (placebo switched to FIL100 group) (all n = 1).

Supplementary Table S1. Baseline disease activity (overall population)

DAS28-CRP, *n* (%)	FIL200 (*n* = 475)	FIL100 (*n* = 480)	ADA (*n* = 325)	PBO (*n* = 475)	Total (*N* = 1755)
≤3.2	2 (0.4)	1 (0.2)	2 (0.6)	0	5 (0.3)
>3.2 to ≤5.1	104 (21.9)	121 (25.2)	72 (22.2)	128 (26.9)	425 (24.2)
>5.1	369 (77.7)	358 (74.6)	251 (77.2)	347 (73.1)	1325 (75.5)

ADA: adalimumab; DAS28-CRP: Disease Activity Score in 28 joints using C-reactive protein; FIL100/200: filgotinib 100/200 mg; PBO: placebo.

Supplementary Table S2. Duration of response

Median (95% CI) duration of response, weeks	FIL200 (*n* = 104)	FIL100 (*n* = 121)	ADA (*n* = 72)	PBO* (*n* = 128)
DAS28-CRP <2.6	15 (11, 31)	7 (5, 9)	9 (5, 13)	5 (5, 7)
CDAI remission (≤2.8)	7 (5, 13)	9 (7, 13)	13 (7, 29)	5 (5, 7)
DAS28-CRP LDA (≤3.2)	28 (13, –)	13 (9, 29)	35 (17, –)	7 (5, 11)
CDAI LDA (≤10)	33 (13, –)	21 (13, 33)	41 (15, –)	9 (7, –)
ACR20	23 (13, 49)	27 (13, –)	27 (15, –)	9 (7, –)
ACR50	17 (9, 41)	9 (7, 13)	15 (11, 33)	7 (5, 9)
ACR70	11 (7, –)	7 (5, 11)	7 (5, 13)	9 (7, –)

*At week 24, placebo-treated patients were re-randomized (1:1) to FIL200 or FIL100 and continued MTX. Patients in the FIL200, FIL100 and ADA groups were treated up to week 52.

Upper CIs are missing for outcomes where they could not be estimated as their corresponding curves remained above 0.5.

Non-responder imputation was applied, meaning that participants with missing outcome data were set as non-responders.

Duration (weeks) of response was calculated as end (weeks) of response – start (week) of response + 1.

ACR20/50/70: 20%/50%/70% improvement in American College of Rheumatology criteria; ADA: adalimumab; CDAI: Clinical Disease Activity Index; CI: confidence interval; DAS28-CRP: Disease Activity Score in 28 joints using C-reactive protein; FIL100/200: filgotinib 100/200 mg; LDA: low disease activity; PBO: placebo.
